# Diagnostic accuracy of in-house real-time PCR assay for *Mycobacterium tuberculosis*: a systematic review and meta-analysis

**DOI:** 10.1186/s12879-019-4273-z

**Published:** 2019-08-08

**Authors:** Zhenhong Wei, Xiaoping Zhang, Chaojun Wei, Liang Yao, Yonghong Li, Xiaojing Zhang, Hui Xu, Yanjuan Jia, Rui Guo, Yu Wu, Kehu Yang, Xiaoling Gao

**Affiliations:** 1 Blood Transfusion Department, Gansu Provincial Hospital, Lanzhou, 730000 Gansu China; 2 The Institute of Clinical Research and Translational Medicine, Gansu Provincial Hospital, No. 204, Donggang street, Chengguan district, Lanzhou, 730000 Gansu China; 3 The Institute of Clinical Study and Evidence-Based Medicine, Gansu Provincial Hospital, Lanzhou, 730000 Gansu China; 4 Evidence-Based Medicine Center and Key Laboratory of Evidence-Based Medicine and Knowledge Translation of Gansu Province, College of Basic Medicine, Lanzhou University, No. 199, Donggang street, Chengguan district, Lanzhou, 73000 Gansu China

**Keywords:** Tuberculosis, Laboratory diagnosis, In-house real-time PCR, Meta-analysis, Systematic review

## Abstract

**Background:**

In recent years, studies on the diagnostic accuracy of in-house real-time PCR (hRT-PCR) assay for the detection of *Mycobacterium tuberculosis* (*Mtb*) have been reported with unignorable discrepancies. To assess the overall accuracy of the hRT-PCR assay for *Mtb* diagnosis in different samples for individuals with active pulmonary and extra-pulmonary *Mtb* infection, a systematic review and meta-analysis were performed.

**Methods:**

The PUBMED, EMBASE, Web of Science, and Cochrane databases were searched up to June 2017 for eligible studies that estimated diagnostic sensitivity and specificity with the hRT-PCR assay in respiratory and non-respiratory samples in pulmonary and extra-pulmonary *Mtb* infection patients, with *Mtb* culture as the reference standard. Bivariate random effect models were used to provide pooled estimation of diagnostic accuracy. Further, subgroup and meta-regression analyses were performed to explore sources of heterogeneity. The risk of bias was assessed by the QUADAS-2 tool.

**Results:**

Of the 3589 candidate studies, 18 eligible studies met our inclusion criteria. Compared to *Mtb* culture data, the pooled sensitivity and specificity were 0.96 and 0.92, respectively. The diagnostic odds ratio (DOR) was 192.96 (95% CI 68.46, 543.90), and the area under the summary ROC curve (AUC) was 0.9791. There was significant heterogeneity in sensitivity and specificity among the enrolled studies (*p* < 0.001). The studies with high-quality assessment and application of respiratory specimen were associated with better accuracy.

**Conclusions:**

In low-income/high-burden settings, our results suggested that the hRT-PCR assay could be a useful test for the diagnosis of TB with high sensitivity and specificity.

**Electronic supplementary material:**

The online version of this article (10.1186/s12879-019-4273-z) contains supplementary material, which is available to authorized users.

## Background

Tuberculosis (TB) remains a major public health problem worldwide. In 2015, 10.4 million new cases of TB were reported. Approximately 1.4 million people died of the disease, and there were an additional 0.4 million deaths from co-infected with HIV [1]. Rapid diagnosis and treatment are pivotal for the effective control of TB in clinical practice [2]. Acid-fast staining and *Mtb* culture are classical *Mtb* diagnosis techniques. The acid-fast stain lacks sensitivity, and the culture requires several weeks for incubation [3, 4]. The inherent limitations make it difficult for them to meet the requirement for early diagnostics [5].

Nucleic acid amplification tests (NAATs), such as polymerase chain reaction (PCR), which was developed in 1983, are now a common tool for the rapid diagnosis of many infectious diseases, including TB [6]. To date, some commercial tests, including COBAS TaqMan, Xpert MTB/RIF and the Abbott Real-Time MTB assay, have been used for TB diagnosis [7–9]. However, due to the limited infrastructure and medical resources, many clinical laboratories in suburban areas with high TB burden cannot afford these assays [1]. In-house polymerase chain reaction (hPCR) that uses equipment and reagents available from diverse suppliers in competitive markets may be more affordable, feasible, and sustainable than Xpert MTB/RIF [10]. hPCR is thus becoming popular in these areas. Several regions of the mycobacterial genome, such as IS6110 and 16S rDNA, have been used as targets for assays [11–13]. Meta-analysis of previous studies demonstrated that the diagnostic accuracy of hPCR assays was variable and inconsistent compared with commercial tests [14, 15]. For example, the sensitivity of hPCR for tuberculosis meningitis varied between 0 and 100% [16]. In recent years, PCR technologies have improved markedly with the development of RT-PCR for the detection of mycobacterial infection [17]. This method has the advantage over conventional PCR in speed, automation, high sensitivity and specificity, and a low risk of cross-contamination [18, 19]. In contrast to the commercial kits, an inexpensive RT-PCR would be particularly popular in regions that are short of medical equipment, such as Brazil, India, China, the Russian Federation, Southeast Asian, South Africa, and East Africa. Medical resources are limited in the majority of these countries [1]. Although recent studies have revealed that RT-PCR assays have good diagnostic performance for TB, there are unignorable discrepancies between their results [10, 20–32]. Moreover, none of the researchers could demonstrate precise diagnostic accuracy due to their limited statistical power. Therefore, by systematic review and meta-analysis, we explored factors associated with heterogeneity as well as diagnostic accuracy of the hRT-PCR assay for TB using data from previous studies.

## Methods

The current meta-analysis was conducted according to the guidelines of the Preferred Reporting Items for Systematic Reviews and Meta-Analyses (PRISMA) statement [33]. Since the study was a systematic review and meta-analysis of published articles, patient consent or approval from the institutional ethics committee was not necessary.

### Search strategy

We searched the following databases: PUBMED, EMBASE, Web of Science and Cochrane Library. All searches were up to date as of June 2017. The search terms used included “tuberculosis”, “*Mycobacterium tuberculosis*”, “nucleic acid amplification techniques”, “real time PCR”, “quantitative real-time polymerase chain reaction”, “PCR, quantitative real-time”, “quantitative real-time PCR”, “real-time PCR, quantitative”, “sensitivity and specificity”, or “predictive value”. In addition, the references of several previously published reviews on NAATs were searched for possible candidate articles.

### Study selection

We included all available studies that reported the assessment of hRT-PCR assay for direct detection of TB. Reasons for studies exclusion were (i) the reference standard was not culture proven *Mtb*; (ii) studies performed with other assays other than hRT-PCR assay (in mixed Methods Research, data were analysed for the eligible cases separately); (iii) application of hRT- PCR assay for determining drug resistance; (iv) incomplete data (lacking any of the availability data including true-positive, true-negative, false-positive and false-negative or these variables could not be calculated from the published data) were not extracted; (v) evaluation of hRT- PCR assay on animal specimens; and (vi) conference abstracts, letters, case reports, editorials, and reviews without original data were excluded.

Two investigators (LY and YHL) independently screened candidate literature by looking up the title and abstract. Then, the full texts of the potentially relevant articles were carefully read to determine whether they could be included. Disagreements were resolved by consensus between the two investigators.

### Data extraction and quality assessment

Two investigators (LY and YHL) independently extracted accurate information from the ultimately included articles. Disagreements were resolved by consensus between the two investigators. The quality of the included studies was independently estimated by two investigators (HX and RG) using a Revised Tool for the Quality Assessment of Diagnostic Accuracy Studies (QUADAS-2), which consists of seven domains [34]. A study was treated as a high-quality study when it had no domain with a high risk of bias and no domain with high applicability concerns.

### Statistical analysis

Analysis was performed using Meta-Disc (version 1.4) software [35]. We pooled the data with the DerSimonian-Laird random effects model (REM), with the following pooled estimates: sensitivity, specificity, positive likelihood ratio (LR+), negative likelihood ratio (LR-), and the diagnostic odds ratio (DOR).

Each study in the meta-analysis contributed a pair of numbers: sensitivity and specificity. A summary receiver operating characteristic (SROC) curve was constructed for the hRT-PCR assay [36]. A shoulder-like curve illustrates that the variability between studies may be due to the threshold effect. A non-shoulder-like curve indicates that sensitivity and specificity are not correlated. The overall diagnostic performance of that hRT-PCR assay was assessed as the area under the curve (AUC) (an AUC value of 100% indicates a perfect test, while an AUC of 50% signifies poor diagnostic accuracy) [37, 38].

Heterogeneity between included studies refers to a high degree of variability in study results. The heterogeneity could be explained by variability in thresholds or differences in test methods and study characteristics. Chi-square and Fisher’s exact tests were used to detect statistically significant heterogeneity. Heterogeneity between included studies was evaluated with subgroup (stratified) analysis and meta-regression analysis [39]. In the subgroup analysis, we computed pooled DOR estimates in various strata. The following factors as potential sources of heterogeneity: study design, target sequence, respiratory specimen versus non-respiratory specimen, the distribution of TB, and components of study quality.

The meta-regression model produces relative diagnostic odds ratios (RDOR) as the output [39]. An RDOR is a ratio of two DORs. An RDOR of 1.0 explains that a particular covariate does not affect the overall DOR. An RDOR> 1.0 explains that studies with a particular characteristic have a higher DOR than studies without this characteristic. For an RDOR< 1.0, the converse is true.

Finally, since publication bias is an important focus for meta-analyses of diagnostic studies [40], the potential publication bias of included studies was assessed by Deeks’s funnel plot (Stata version 12.0; Stata Corp., College Station, TX).

## Results

### Study search

**Fig. 1:**
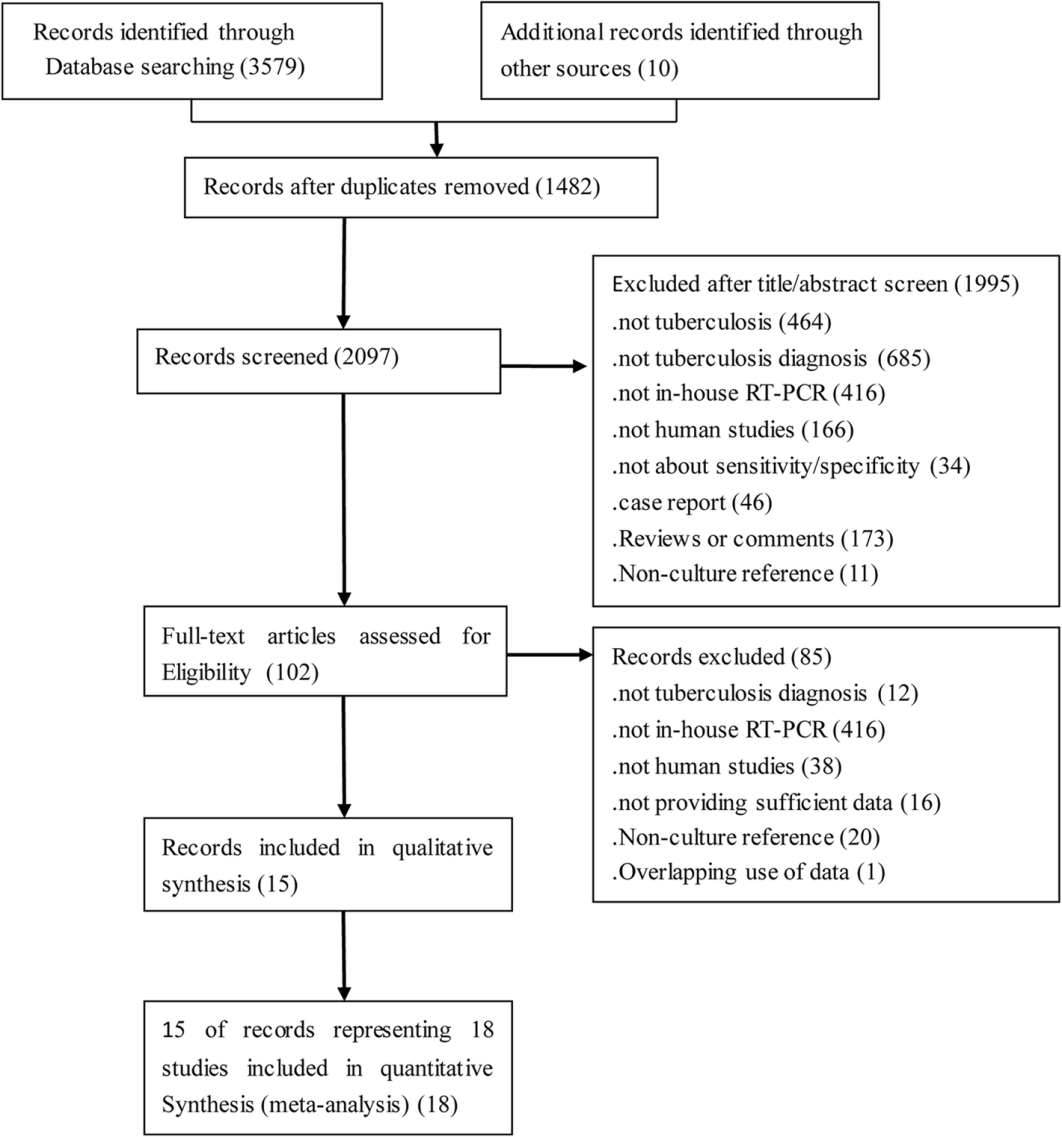
The study search flow chart

Of the 3589 unique articles, we finally identified 15 eligible articles representing 18 independent studies (Fig. 1). The performance of the hRT-PCR assay in *Mtb* detection of clinical specimens was evaluated from all included studies with *Mtb* culture as a reference standard. Summary characteristics of the included studies are shown in Table 1. Eleven studies used respiratory specimens, and five used non-respiratory specimens. Two studies focused on patients with HIV-associated TB. Five studies were from Brazil, two were from India, and the remaining studies were from eight different countries. Among them, eight are the high TB burden countries. Eleven studies used IS6110 as an amplification target, and 7 studies used other targets (e.g., mpt64 and senX3-regX3). A total of 3281 samples, including 2809 respiratory samples and 472 non-respiratory samples, provided valid results.

**Table 1:** Characteristics of the included studies

Author Year	Study design	Country	Number of participants	Number of samples sent for culture	Culture+/−	Type of test (target sequence)	Acid-fast stain	Culture	Respiratory specimen	Non-respiratory specimen	R/NR	TP	FP	FN	TN
Aggarwal 2012 [20]	Cross-sectional	India	80	80	18/62	16 s rRNA	?	MGIT	–	csf	0/80	12	14	6	48
Albuquerque 2014 [21]	Cross-sectional	Brazil	140	140	47/93	IS6110	ZN	7H9,LJ	sp	–	140/0	41	1	6	92
Barletta 2014 [10]	Cross-sectional	Peru	112	112	84/28	IS6110	ZN	LJ	sp	–	109/0	79	1	5	25
Chaidir 2013 [22]	Case-control	Indonesia	230	230	102/105	IS6110	ZN	Liquid and solid	–	csf	0/207	94	46	8	59
Darban-Sarokhalil 2012 [23]	Case-control	Iran	247	247	112/135	cyp141	ZN	LJ	sp	–	247/0	101	3	11	132
Gallo 2016 [24]	Cross-sectional	Brazil	?	1451	1351/100	mpt64	?	Liquid and solid	sp	–	1451/0	1347	4	4	96
Inoue 2011 [25]	Cross-sectional	Singapore	414	414	55/128	IS6110	?	MGIT	sp	csf,pf,ti	104/66	43	3	8	116
Lee 2011 [26]	Case-control	Korea	370	129	53/76	senX3-regX3	ZN	3% Ogawa	ti	ti	53/76	47	1	16	65
Lira, LA 2012 [27]	Case-control	Brazil	165	165	66/99	IS6110	ZN	LJ	sp	–	165/0	58	2	8	97
Lyra 2014 [28]	Cross-sectional	Brazil	181	194	11/91	IS6110	ZN	LJ	sp	–	102/0	11	3	0	88
Miller 2011 [29]	Cross-sectional	America	90	112	89/23	IS6110	?	MGIT, LJ,7H11	sp,ba,bal.,ti	In,ab,pf,ti	89/23	30	4	7	71
Rao 2016 [30]	Cross-sectional	India	100	200	44/56	16sRNA	?	MGIT	sp	–	100/0	44	2	0	54
Rozales 2014 [31]	Cross-sectional	Brazil	447	447	42/405	IS6110	ZN	7H9,MGIT	sp,bal	–	124/0	41	7	1	75
Sanjuan-Jimenez 2015 [32]	Case-control	Spain	153	145	76/69	senX3-regX3	ZN	LJ,MGIT	sp,ba,bal	pf,In,ur,csf,ar	125/20	65	0	11	69
Sanjuan-Jimenez 2015 [32]	Case-control	Spain	153	145	76/69	IS6110	ZN	LJ,MGIT	sp,ba,bal	pf,In,ur,csf,ar	125/20	72	9	4	60

Acid-fast stain: ZN, Ziehl-Neelsen; Culture: MGIT, Mycobacteria growth indicator tube; LJ, Löwenstein-Jensen; 7H9, Middlebrook 7H9 Broth. Respiratory specimen: sp., sputum; ba, broncheal/tracheal aspirate; bal., bronchialalveolar lavage; ti, tissue specimen. Non-respiratory specimen: ln, lymph node; pf, pleural fluid; ar, articular fluid; ab, abcess/pus; ur, urine; csf, cerebrospinal fluid; ti, tissue sample. Specimen number: R, number of the respiratory specimens; NR, number of the non-respiratory specimens. “?” represents that the specific method is not mentioned in this paper. TP, true-positive; FP, false-positive; FN, false-negative; TN, true-negative

### Quality evaluation

**Fig. 2:**
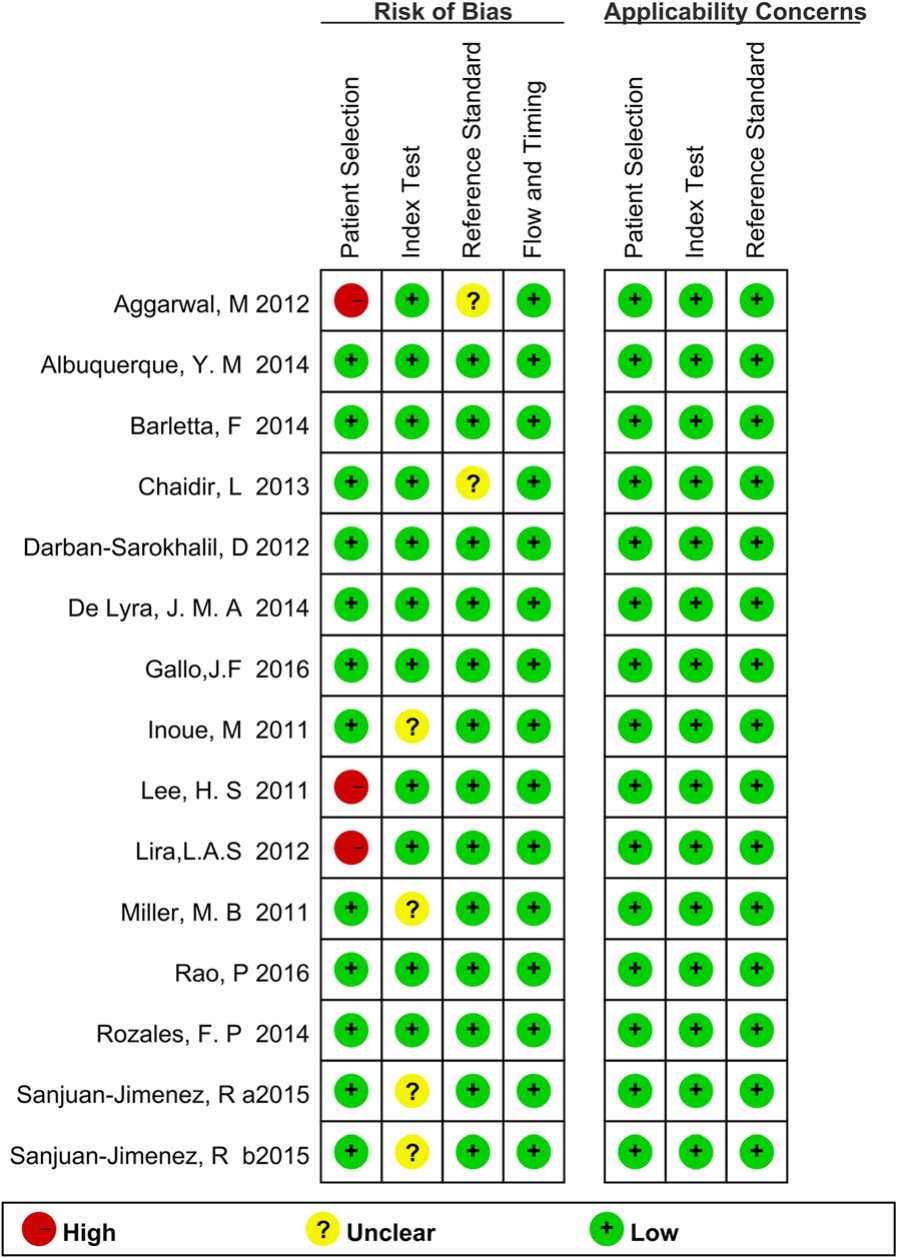
Summary of methodological quality of studies according to the QUADAS-2 (Quality Assessment of Diagnostic Accuracy Studies-2) tool. High-quality study: a study that had no domain with a high risk of bias and no domain with high applicability concerns; medium/moderate-quality study: a study that had domain with an unclear risk of bias or domain with unclear applicability concerns; low-quality study: a study that had a domain with a high risk of bias and domain with high applicability concerns

We assessed the quality of the studies by QUADAS2. To assess the risk of bias regarding patient selection, three studies were deemed to be of case-control design, which compared diagnosed TB patients to non-TB individuals. Therefore, there was a high risk of bias based on the patient selection method after QUADAS2 assessment. As for “index test” evaluation, four studies failed to illustrate the blind working flow. Given the advanced acknowledgement of the reference test results, the bias could arise from the interpretation of the index test. These studies had an unclear risk of bias on the index test. For the reference standard, two studies did not provide sufficient description concerning the reference test results. Therefore, these studies were considered to have an unclear risk of bias on the reference test. No other domain had a high risk of bias or a high applicability concern (Fig. 2).

### Diagnostic accuracy of hRT-PCR assay

**Fig. 3:**
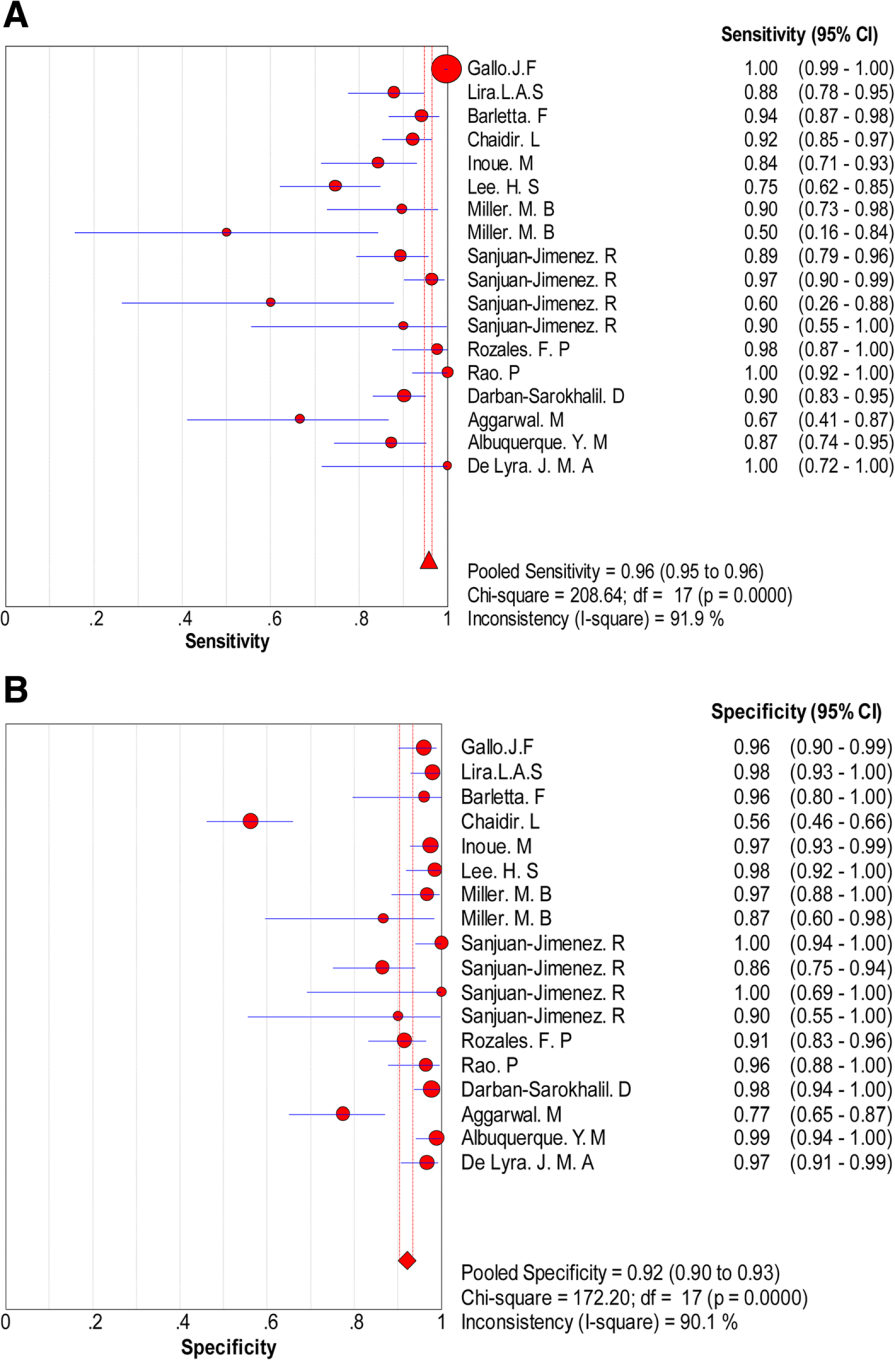
Forest plot of specificity and sensitivity estimates. **a** Forest plot of sensitivity estimates and 95% confidence intervals (CI). The point estimates of sensitivity from each study are shown as solid circles. Error bars are 95% CI. Circles are proportional to study size. The pooled estimate is denoted by the diamond at the bottom. **b** Forest plot of specificity estimates and 95% CI. The point estimates of specificity from each study are shown as solid circles. Error bars are 95% CI. Circles are proportional to study size. The pooled estimate is denoted by the diamond at the bottom

**Fig. 4:**
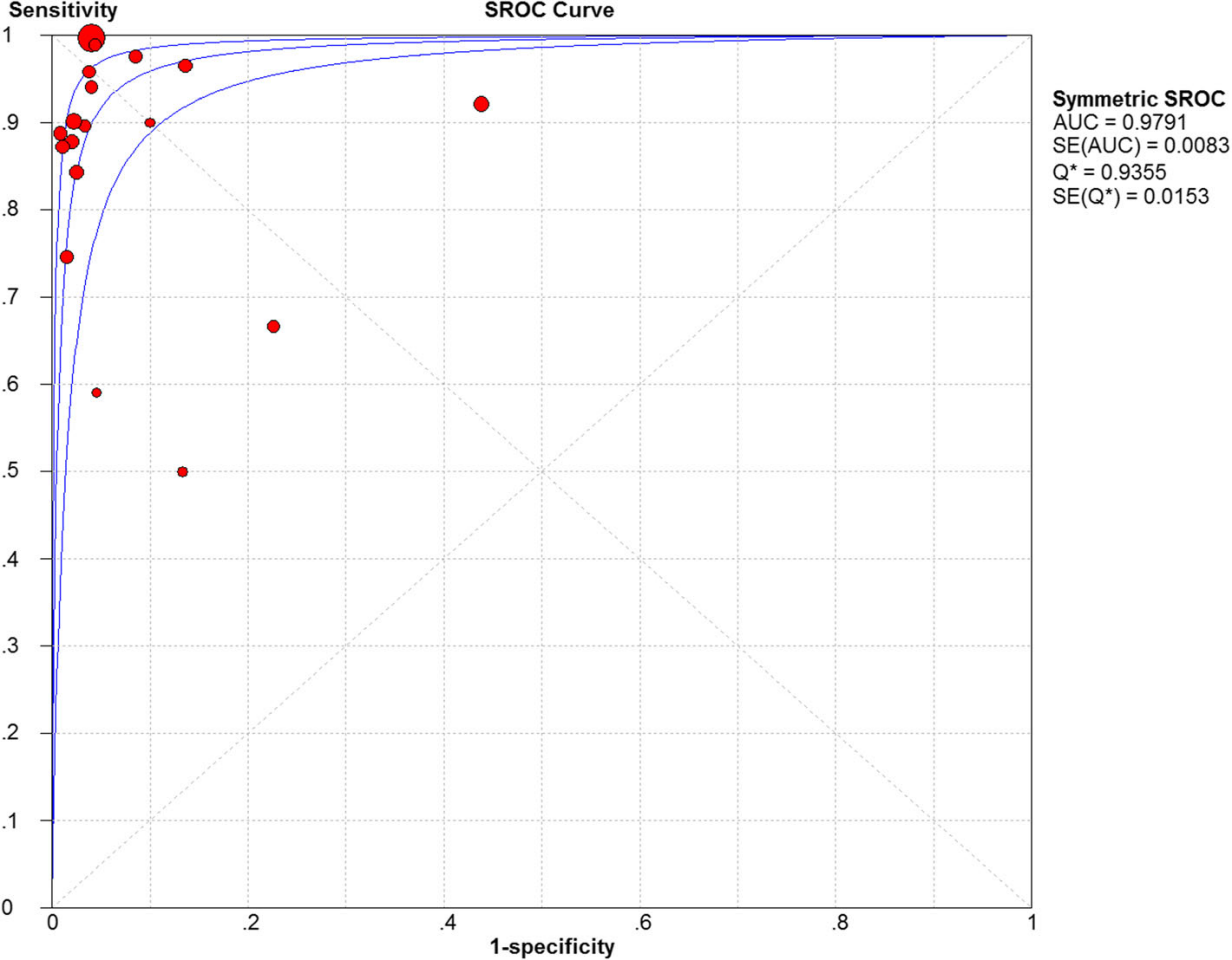
Summary receiver operating characteristic curves for RT-PCR assays. Each solid circle represents each study in the meta-analysis. The curve is the regression line that summarizes the overall diagnostic accuracy. SROC = summary receiver operating characteristic; AUC = area under the curve; SE (AUC) = standard error of AUC; Q* = an index defined by the point on the SROC curve where the sensitivity and specificity are equal, which is the point closest to the top-left corner of the ROC space; SE (Q*) = standard error of Q* index

When all 18 studies using the hRT-PCR assay were evaluated together, the overall sensitivity and specificity estimates were 0.96 (95% CI 0.95, 0.96) and 0.92 (95% CI 0.90, 0.93), respectively. The sensitivity and specificity of all studies are shown in the forest plot (Fig. 3a, b). The overall LR+ was 16.90 (95% CI 7.22, 39.56), and LR- was 0.11 (95% CI 0.06, 0.18). The pooled DOR was 192.96 (95% CI 68.46, 543.90). Heterogeneity was detected by chi-square analysis in the summary results. All measurements showed high heterogeneity (*p* < 0.001 for the test of heterogeneity). The accuracy was measured, and their corresponding chi-square test was applied to statistically analyse heterogeneity (Table 2). The overall accuracy of the hRT-PCR assay in a summary receiver operating characteristic (SROC) curve is displayed in Fig. 4, and the curve displayed a trade-off between sensitivity and specificity. The area under the SROC curve (AUC) was 0.9791, indicating a highly diagnostic accuracy. Overall, significant heterogeneity in sensitivity and specificity deserves more attention in the clinical applications of the hRT-PCR assay in TB detection.

**Table 2:** Pooled Summary Estimates of All Studies

Accuracy Measure	Pooled summary measure^a^ (95% CI)	*P* value for heterogeneity^b^
Sensitivity	0.96 (0.95–0.96)	< 0.001
Specificity	0.92 (0.90–0.93)	< 0.001
Positive Likelihood Ratio (LR+)	16.90 (7.22–39.56)	< 0.001
Negative Likelihood Ratio (LR-)	0.11 (0.06–0.18)	< 0.001
Diagnostic Odds Ratio (DOR)	192.96 (68.46–543.90)	< 0.001

^a^Random effects model

^b^Chi-square or Fisher’s exact test for heterogeneity

### Exploration of heterogeneity

Heterogeneity is an important concern on diagnostic meta-analysis. The threshold effect, method differences and study characteristics may lead to the variability. The SROC curve with studies was weighted by their inverse variance, as shown in Fig. 4. The non-shoulder-like curve indicated no threshold effect in the current meta-analysis. Furthermore, the Spearman correlation coefficient was 0.147, and the *p* value was 0.562. It illustrated no threshold effect. Subgroup analysis was also used to explore other factors that were associated with heterogeneity by stratifying data into relatively more homogeneous strata. The DOR estimates of the study characteristics are compared in Table 3. The heterogeneity could be explained in some strata, including specimen type, the distribution of TB, and quality of studies. However, even after stratification, the heterogeneity remained in the evaluation of diagnostic accuracy.

**Table 3:** Stratified analyses for the evaluation of heterogeneity in studies with real-time PCR assay

Subgroup (Number of studies)	Summary diagnostic odds ratio (95% CI)^a^	Chi^2^ square test of heterogeneity	*P* value for heterogeneity^b^
Study design			
Cross-sectional (10)	403.18 (120.05–1354.05)	36.66	< 0.001
Case-control (8)	73.86 (20.40–267.48)	34.01	< 0.001
Target sequence			
IS6110 (11)	144.74 (51.24–408.86)	39.39	< 0.001
Other target (7)	297.17 (30.22–2921.73)	66.27	< 0.001
Specimen type			
Respiratory (11)	598.12 (269.12–1329.32)	19.09	0.039
Non-respiratory (5)	12.39 (6.67–22.73)	3.57	0.468
Both (2)	202.47 (64.68–633.83)	0.00	0.944
Region of study			
TB high-burden country (8)	281.86 (37.69–2107.75)	90.46	< 0.001
Other country (10)	160.73 (72.80–354.83)	15.17	0.086
Quality of study			
High-quality (7)	926.97 (303.59–2830.38)	12.83	0.046
Medium-quality (8)	76.77 (22.98–256.50)	26.65	< 0.001
Low-quality (3)	72.35 (4.47–1170.04)	19.07	< 0.001

^a^Random effects model

^b^chi-square or Fisher’s exact test for heterogeneity; high-quality study: a study that had no domain with a high risk of bias and no domain with high applicability concerns; medium/moderate-quality study: a study that had domain with a unclear risk of bias or domain with unclear applicability concerns; low-quality study: a study that had a domain with a high risk of bias and domain with high applicability concerns

**Fig. 5:**
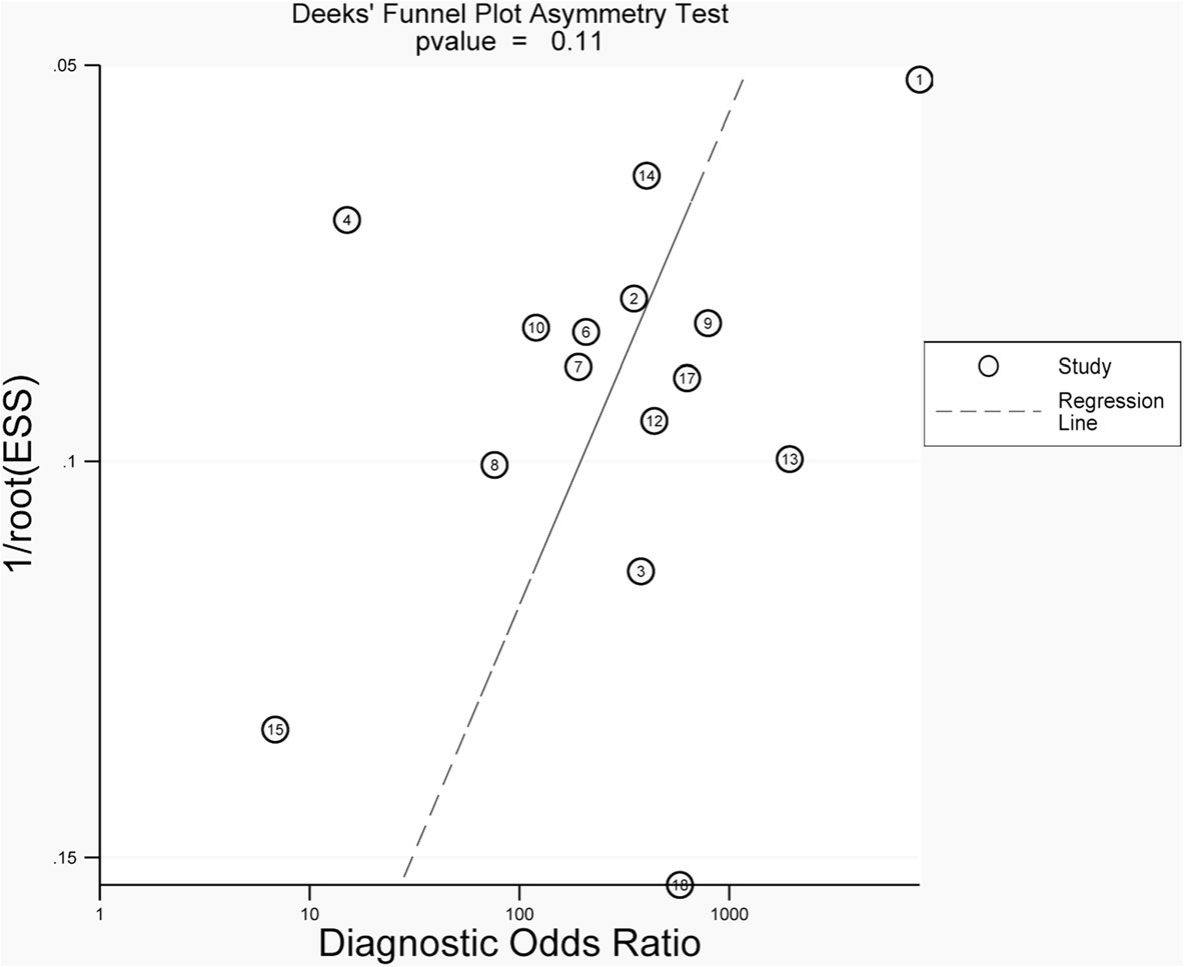
A Deeks’ funnel plot assessment test evaluated the potential publication bias for in-house RT-PCR assays. The plot shows the symmetric distribution of the log of diagnostic odds ratios against the inverse root of effective sample sizes (ESS), indicating the absence of any publication bias

We further performed a meta-regression analysis to explain the variation after subgroup analysis. As shown in Table 4, the RDOR was established from the meta-regression analysis using the restricted maximum likelihood (REML) method to measure between-study variance. Studies with respiratory specimens produced RDOR values that were significantly higher than those used non-respiratory specimens or both specimens. Studies with a high-quality level produced RDOR that were significantly higher than those with medium quality levels or low-quality levels. The distribution of TB displayed a slightly higher RDOR but no statistical significance in the final regression model. Study design and target sequence did not produce a significant RDOR, indicating that the use of any study design and target sequence did not substantially affect diagnostic accuracy. Therefore, specimen types and quality of studies may affect accuracy heterogeneity. Evaluation of the Deeks’ (*p* = 0.11) test did not show evidence of publication bias. Furthermore, the funnel plot did not display the presence of asymmetry (Fig. 5).

**Table 4:** Meta-regression analysis to determine sources of heterogeneity

Intercept	Coefficient	*P* value	Relative diagnostic odds ratio (RDOR)	95% confidence interval
Intercept	5.347	0.0000	–	–
Threshold (S)	0.169	0.5382	–	–
TB high-burden country vs. other country	0.756	0.3056	2.13	(0.46;9.96)
IS6110 vs. other target sequences	−0.812	0.2266	0.44	(0.11;1.77)
Cross-sectional design vs. case-control design	−0.759	0.5102	0.47	(0.04;5.45)
High-quality level vs moderate/low-quality level	1.175	0.0272	3.24	(1.17;9.00)
Respiratory specimens vs non-respiratory specimens /both	2.262	0.0025	9.60	(2.54;36.25)

High-quality study: a study that had no domain with a high risk of bias and no domain with high applicability concerns; medium/moderate-quality study: a study that had domain with an unclear risk of bias or domain with unclear applicability concerns; low-quality study: a study that had a domain with a high risk of bias and domain with high applicability concerns

## Discussion

### Principal findings

We summarized the evidence on the accuracy of the hRT-PCR assay for the diagnosis of TB and performed a meta-regression analysis to explore factors involved in in-house RT-PCR assay performance. This meta-analysis included 18 independent studies with a total of 97% AUC, indicating that the hRT-PCR assay for TB detection was useful in rapidly identifying TB cases and that negative data guaranteed the certainty for ruling out active TB. Since there is significant performance heterogeneity in our recruited studies, subgroup and meta-regression analysis indicated that the use of respiratory specimens and studies with high quality were associated with better diagnostic accuracy of hRT-PCR.

### Clinical implications

Even though the meta-analysis shows the power in evaluating the overall diagnostic accuracy of hRT-PCR, more caution is necessary to determine clinical accuracy due to significant heterogeneity. Previous meta-analyses [14, 16, 41] did not fully interpret the cause of heterogeneity found in hPCR results across studies. Our results showed that respiratory specimens and high-quality design were associated with better diagnostic accuracy of the hRT-PCR assay, which was consistent with a recent meta-analysis of the Xpert MTB/RIF PCR assay for the diagnosis of extra-pulmonary TB. There was a performance difference in the specimen site, with low sensitivity in pleural fluid (37%) and cerebrospinal fluid samples (69%) [42]. This finding was not surprising given the paucibacillary nature of these specimens documented in other studies and meta-analysis [14, 16, 42]. The “case-control” study design and the IS6110 targeted sequence for hPCR were associated with better accuracy based on previous empirical research and meta-analyses [14, 43–45]. Some researchers were concerned that the case-control study might overestimate the diagnostic accuracy since it samples patients from the extreme ends of the clinical spectrum (an ideal, “extreme contrast” setting). For example, the sensitivity of a test is evaluated in seriously diseased subjects, and the specificity in healthy individuals [46]. In our meta-analyses, laboratory factors (such as target sequence and amplification technique) weighted more on accuracy than study design features.

The IS6110 gene was widely used for both pulmonary and extra-pulmonary TB diagnosis [13, 47, 48]. Due to its multiple copies in the genome of the *Mtb* complex, PCR might result in better sensitivity [14]. However, our data demonstrated that study design with IS6110 had little impact on diagnostic accuracy. This is possible because RT-PCR used in our enrolled studies carries better advanced technology compared to conventional PCR. RT-PCR uses built-in automated thermocyclers and fluorimeters to monitor PCR reactions in a single tube format in which the reaction processes rapidly and minimizes the risk of contamination from product carryover [49]. Therefore, RT-PCR can provide reliable and repeatable results.

The performance of hRT-PCR was heterogeneous across studies; some patients could have false-positive hRT-PCR results and others false negative. Accuracy is related to the standard/reference assay, TB culture. Reliability is based on clinical diagnosis of TB disease. However, not all recruited studies have evaluated their hRT-PCR according to these standards. Caution is highly necessary for the clinical implications and applicability of hRT-PCR. The combination with other clinical information, such as the disease history, family medical records, microscopy screening and histopathology data, is recommended in clinical practice.

### Previous meta-analyses of nucleic acid amplification (NAA) test accuracy

PCR technology is widely used in the diagnosis of infectious diseases. Multiple commercial and in-house NAA techniques have been developed for TB diagnosis [41, 50]. Xpert MTB/RIF, approved by the WHO and the FDA, is a novel, rapid, automated, cartridge-based NAA test that can simultaneously detect TB and rifampicin resistance directly on untreated sputum [51]. In addition, Xpert MTB/RIF was recommended for the diagnosis of TB in some special subjects, such as children and patients with certain forms of extra-pulmonary TB. A systematic review showed that Xpert MTB/RIF offered an acceptable sensitivity (62%) and specificity (98%) for the diagnosis of pulmonary tuberculosis in children [52]. Compared to hRT-PCR, the main features of Xpert MTB/RIF are its ability to detect resistance to rifampicin with a simple procedure and high cost. Xpert MTB/RIF may be practical for middle/high income regions. In fact, the majority of low- and middle-income countries, particularly those with limited resources, smear microscopy was still used for TB diagnosis [52]. Therefore, the hRT-PCR assay might substitute Xpert MTB/RIF in a low-income setting where multi-drug resistant TB is not prevalent. Another practical assay that might meet the needs of urban areas is loop-mediated isothermal amplification (LAMP) with a commercialized LAMP kit (Loopamp MTBC) [53]. A previous systematic review concerning the LAMP assay included 27 studies [54]; 9 out of the 27 studies evaluated the Loopamp MTBC, and the other 18 evaluated in-house LAMP assays. The summary of sensitivity and specificity for Loopamp MTBC were 80.9 and 96.5%, versus 93.0 and 91.8% for in-house LAMP assays, respectively. LAMP seems inferior to the RT-PCR tests in our analysis. Considering their low cost and simplicity, LAMP assays might be accepted in countries with limited resources.

### Limitations of the review

Our review had some limitations. First, only one study evaluated the diagnostic test accuracy of the hRT-PCR assay for smear status, and only two studies included HIV-positive patients. Therefore, we could not determine the effect of smear and HIV status on the accuracy of the hRT-PCR assay. Second, we only included published studies in English, and this could have caused bias in our conclusion. Third, despite the fact that the subgroup analysis and meta-regression analysis could explain part of the observed heterogeneity in accuracy estimates, considerable heterogeneity remained unexplained. Finally, although we searched as many sources as possible, some eligible studies may have been missed.

## Conclusion

In conclusion, based on the meta-analysis using the bivariate model, the diagnostic accuracy of the hRT-PCR assay for TB detection was acceptable. Subgroup and meta-regression analyses were performed, and we found that the diagnostic characteristics were different, depending on the specimen type and quality of the studies. Thus, the hRT-PCR assay, a relatively inexpensive assay compared to other commercial kits, has potential practical value for diagnosing TB, especially in low-income/high-burden settings, where infrastructures and medical resources are limited.

## Additional file

Additional file 1: Raw data. (XLSX 11 kb)

## Data Availability

The dataset supporting the conclusions of this article is included within the article’s additional file (Additional file 1).

## Abbreviations

AUC: Area under the curve
CI: Confidence interval
DOR: Diagnostic odds ratio
hPCR: In-house polymerase chain reaction
hRT-PCR: In-house real-time polymerase chain reaction
LAMP: Loop-mediated isothermal amplification
Mtb: *Mycobacterium tuberculosis*
NAATs: Nucleic acid amplification tests
NLR: Negative likelihood ratio
PLR: Positive likelihood
PRISMA: Preferred reporting items for systematic
QUADAS: Quality Assessment of Diagnostic Accuracy Studies
RDOR: Relative diagnostic odds ratios
REM: Random effects model
SROC: Summary receiver operating characteristic
TB: Tuberculosis
